# Moisture-driven local strain development in ASR-damaged concrete

**DOI:** 10.1371/journal.pone.0347208

**Published:** 2026-06-01

**Authors:** Seren Azad, Pavel Trtik, Markus Strobl, Anders Kaestner

**Affiliations:** PSI Center for Neutron and Muon Sciences, Villigen PSI, Switzerland; University of Sharjah, UNITED ARAB EMIRATES

## Abstract

Although alkali–silica reaction (ASR) is widely recognized as one of the major concrete durability issues, the mesoscale response of pre-existing ASR products to moisture exposure remains poorly understood. In this study, neutron tomography combined with image registration is employed to non-destructively quantify moisture-induced local deformation in concrete specimens already affected by ASR damage. Three concrete mixes with different aggregate reactivities were imaged before and after 72 hours of water exposure. The resulting volumetric strain fields show that the largest localized expansions are concentrated within the crack network of the cement paste, where amorphous ASR products are expected to be prevalent. In contrast, cracks within aggregates, which typically contain crystalline ASR phases, exhibit substantially lower expansion. These observations provide direct experimental evidence supporting the hypothesis that amorphous ASR products possess a greater swelling potential than crystalline counterparts.

## 1 Introduction

As a primary construction material, concrete underpins the development of modern infrastructure worldwide [1]. However, the durability of concrete is compromised by several physicochemical processes, among which the alkali-silica reaction (ASR) [2] has emerged as a significant concern for engineers and researchers. Although ASR was first identified in the 1940s, a full understanding of its complex mechanisms including initiation and how its already-formed (solidified) products interact with water (further propagation of damage), remains elusive. This challenge is largely due to its complex, multi-scale, and time-intensive nature [2]. More specifically, some of the factors that make ASR research challenging and limit our thorough understanding include:

- **variability in aggregate reactivity**: The reactivity of silica in aggregates can vary widely, depending on the rock type, composition and its geological formation. This variability influences the type, rate and extent of ASR product formation [3];
- **moisture distribution and dynamics**: The role of moisture is critical in ASR processes, including initiation, formation and further propagation. The movement and distribution of moisture within concrete are influenced by environmental conditions and the concrete porosity [4];
- **long-term nature**: ASR in field structures is a gradual process that may take years or decades to manifest as visible damage. Laboratory assessment can be accelerated, and some screening methods provide measurable expansion within days to weeks [5–7]. However, these fast methods often rely on severe conditions, such as elevated temperature or highly alkaline exposure, which may not fully represent field behavior. More representative concrete prism and performance-based tests generally require substantially longer durations [8]. Consequently, conducting real-time, in-situ, non-destructive observations of moisture-driven ASR evolution remains highly challenging [9].
- **multi-scale nature**: Even if in-situ tests were feasible, ASR’s critical processes occur at the nano- to microscale, making them too subtle to capture and quantify directly at all the length scales, in particular the initiation processes [10].

Given these challenges, ASR research has adopted a multifaceted approach that integrates advanced non-destructive and complementary destructive methods to unravel its mechanisms across various length scales [2,3,9,11]. Based on the current understanding, ASR involves a series of chemical reactions between the alkaline components of Portland cement—primarily sodium and potassium hydroxides—and reactive forms of silica, particularly amorphous silica, present in aggregates [3]. In the presence of moisture, the hydroxide ions dissolve the reactive silica, leading to the formation of an alkali-silica solution, which ultimately reacts further to produce hydrated ASR products [2]. These products initially have a high water content and little to no calcium ions (Ca²⁺), and can flow easily as viscous phase that lacks a fully consolidated structure [9]. The Ca² ⁺ available typically in the hydrated cement matrix, alters the chemical equilibrium by lowering the solubility of silica and promoting the formation of a new phase [3]. In essence, Ca²⁺ as cross-linking agents that facilitate the nucleation and growth of a more consolidated ASR product. Depending on the local chemical conditions, this product may develop a moderately crystalline structure—with identifiable basal spacings—or remain poorly crystalline or amorphous, reflecting its complex microstructure. This new solid phase precipitates as a lower-density material, meaning that for a given mass it occupies a larger volume than the original, more concentrated alkali-silica solution. In confined spaces, such as micro-cracks or regions where reactive silica has dissolved, this volumetric increase generates internal pressure, which contributes to crack propagation and the overall expansion observed in ASR-damaged concrete [2,3]. The overall reaction could be represented as:


$$\begin{array}{c} \underset{aggregates}{\underbrace{SiO_{\text{2}}}}+\underset{cement\;pore\;solution}{\underbrace{NaOH/KOH}}+\underset{cement\;pore\;solution}{\underbrace{H_{\text{2}}O}}+\underset{cement\;pore\;solution}{\underbrace{Ca^{2+}}}\to C-A-S-H+etc. \end{array}$$

Here, C-A-S-H stands for Calcium-Alkali-Silica-Hydrate in cement chemistry notation, which represents the complex, hardened ASR products. Based on previous works [9,12,13], it was observed that in the early stages of ASR, the precipitation of solid C-A-S-H within or adjacent to the dissolved portions of aggregates is the primary factor generating expansive (nucleation) pressure that initiates crack formation. As these cracks develop and widen, they expose fresh reactive silica to the alkaline environment, accelerating the reaction in a positive feedback loop. This continuous formation of additional ASR products further amplifies the expansive forces, ultimately contributing to more severe cracking and degradation [9].

One significant gap in our understanding of ASR is how natural wetting and drying cycles influence the long-term evolution and damage in pre-formed ASR products within damaged concrete structures. While many studies have concentrated on the initial reaction mechanisms, such as the dissolution of reactive silica and the early stages of product formation, real-world conditions subject structures to repeated moisture fluctuations. The importance of cyclic moisture exposure is also reflected in performance-based ASR test methods such as RILEM AAR-12 [14], a 60 °C concrete prism test with alkali supply. Compared with RILEM AAR-11 [15], AAR-12 uses modified storage conditions, including cyclic drying, humid storage, and NaCl-solution exposure, to account directly for external alkali exposure such as de-icing salts in pavement environments. However, such tests are primarily designed to assess expansion at the specimen level under accelerated conditions, and they do not directly resolve how moisture fluctuations redistribute strain within pre-existing ASR-damaged mesostructures. These cycles can induce additional processes, including continued silica dissolution, re-precipitation of ASR products, and mechanical microstructural changes in the already formed products. Field observations suggest that these dynamic moisture interactions can accelerate expansion and crack propagation in ways that exceed predictions based solely on product formation models [4,9]. This discrepancy underscores the need for a deeper investigation into how environmental moisture dynamics drive the progression of ASR and ultimately compromise structural durability. Therefore, in the present study we focus on the interaction between water and pre-formed ASR products present in damaged concrete samples with three types of aggregates.

Although the traditional view posits that ASR products [2] (regardless of their crystallographic structure) swell upon water uptake, thereby driving expansion in concrete, this notion has become increasingly controversial. In particular, the moisture response of ASR products depends not only on their crystallographic order but also on composition: higher Na/Si and K/Si ratios generally promote hydrophilicity and free swelling, whereas the influence of Ca/Si is non-linear and can either stabilize or suppress swelling depending on composition and reaction conditions [16]. Recent studies using high-resolution imaging and in-situ analytical techniques have begun to reveal a more complex picture, suggesting that our understanding of moisture’s role in ASR is far from complete [4,10]. In the previous study, in-situ 3D micro-XRD and micro-tomography were used [4] to closely examine ASR products’ moisture response. The ASR-damaged concrete was fractured to extract small aggregate portions exhibiting visible ASR products, and then subjected these samples to controlled humidity variations to monitor their microscale response. Our analysis demonstrated that the ASR products found within the aggregate are predominantly nano-crystalline, exhibiting distinct basal spacings and a heterogeneous spatial distribution. Notably, while one phase showed some increased basal spacing under moist conditions—indicating localized micro-expansion—this did not result in significant macroscopic swelling.

**Fig 1:**
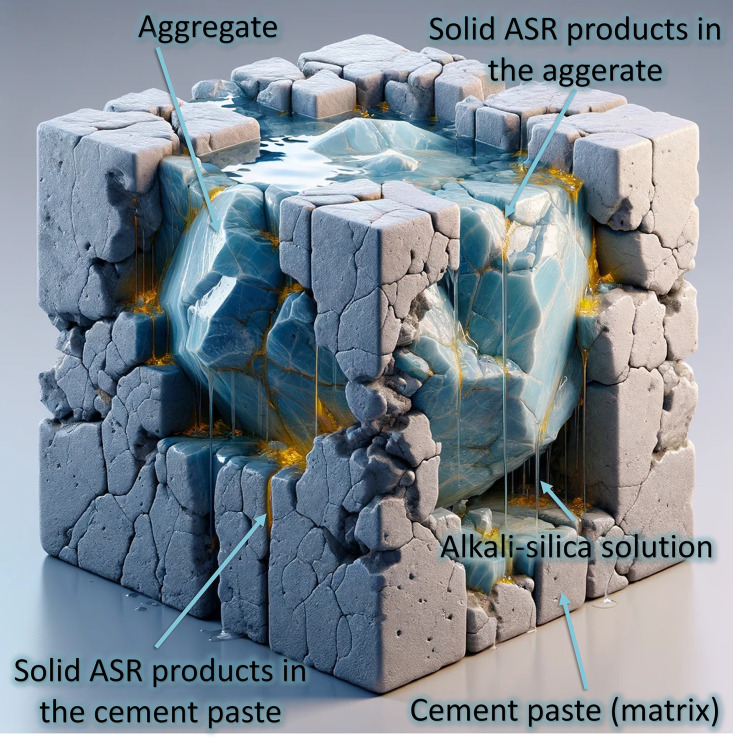
Schematic illustration of alkali-silica (ASR) damage: a hypothetical representation of the currently understood damage development in an aggregate and its surrounding. The schematic illustration was created using OpenAI’s DALL·E, accessed through ChatGPT.

While our previous 3D micro-XRD and micro-tomography studies have provided significant insights into the water interaction of the ASR processes at the microscale, their limited evaluation volume makes it difficult to assess moisture effects at larger scales. Additionally, ASR products forming at the aggregate edges or extending into the cement paste typically do not exhibit a crystalline microstructure. Leemann et al.‘s study [3] on several ASR-damaged aggregates reveals morphological differences between ASR products in aggregates and those in the cement paste: crystalline ASR products are typically found within the aggregates, while a smoother, untextured, and amorphous microstructure predominates at the aggregate-cement interface and within the cement paste. Previous petrographic and microscopy-based studies have shown in two-dimensional sections that ASR products may occur not only within reactive aggregates, but also at aggregate-paste interfaces and within cracks extending into the surrounding cement paste [3,17]. Building on this background, our earlier tomography-based studies provided non-destructive three-dimensional evidence of this redistribution at the mesoscale and enabled its quantitative tracking over time. The application of tomography enables us to study ASR dynamics over a more representative length scale (microns to cm also referred to as meso-scale) and in a non-destructive manner. In the previous research [9] time-lapse X-ray tomography was used to study ASR damage development in concrete samples (also used for the current study) under controlled laboratory conditions (concrete submerged in alkaline solution under 40 °C). Enhanced by contrast agents like BaSO_4_ (i.e., used in cement paste) and CsNO_3_ (i.e., incorporated into the ASR products), this method provided a robust framework for analyzing the evolution of ASR products and crack networks within concrete. We observed that the ASR products, while in the viscos solution state (not precipitated or solidified yet), get transported from aggregates into the cement paste along propagating cracks and can solidify there [3,9,12,13]. This leads to nearly equivalent amounts of ASR products forming within the surrounding cement paste as those forming within the aggregates, revealing that the cement paste, considered less affected, hosts a considerable amount of products. Fig 1 schematically illustrates the ASR-induced damage within an aggregate and its adjacent cement matrix, reflecting our current understanding [9,12,13].

While micro-scale analytical studies suggest differential swelling behavior, direct observational evidence showing the spatial distribution of moisture-induced strains within the complex matrix of aged concrete at a meso-scale has been lacking. In other words, previous investigations into the moisture response of ASR have largely been confined to two distinct scales. At the **macro-scale**, studies have documented the bulk expansion of concrete prisms subjected to varying humidity cycles, establishing a clear link between moisture availability and overall expansion. However, these studies cannot identify the specific locations within the concrete’s mesostructure where this expansion originates. Conversely, at the **micro-scale**, techniques as discussed above, have provided compelling qualitative visualizations of ASR gel swelling on exposed surfaces or as free-standing products. While insightful, these idealized observations do not capture the behavior of ASR products confined within the complex, three-dimensional crack network of a bulk sample. This leaves a critical gap in understanding how localized micro-scale swelling translates into meso-scale strain fields and, ultimately, macroscopic damage.

This study aims to fill that observational gap by using moisture distribution and strain mapping to visualize these interactions. To achieve this, we used neutron tomography [17,18], which is particularly well suited for directly visualizing water while also resolving the crack network and mesostructure of the concrete, similarly to X-ray tomography. We map the volumetric strain development both globally and locally to visualize moisture-induced expansions across a large volume of our concrete samples, observing the behavior in regions typically associated with crystalline and amorphous ASR products. This approach allows for direct observation of the interplay between moisture dynamics and ASR progression at the meso-scale, offering insights that contribute to bridging the gap between microscale reaction mechanisms and macroscale structural degradation [4,9]. The observational insights from our investigation can inform the mechanistic assumptions in computational models of ASR behavior and contribute to the foundational knowledge for developing ASR-resistant concrete formulations and improving predictive maintenance strategies for aging infrastructure [2].

## 2 Materials and methods

### 2.1 Concrete samples

Since the samples used in this study are derived from a previous work (for comprehensive details see [9,12,13]), we only briefly summarize the key aspects here. Three types of concrete specimens were cast using an identical mix design but incorporating distinct aggregates from various regions of Switzerland—labeled “U” for canton Uri, “P” for Praz in canton Valais, and “B” Brienz in canton Bern, respectively. The U aggregates, which consist of sedimentary impure sandstones rich in microcrystalline quartz, are highly reactive. In contrast, the P and B aggregates—both metamorphic (granitic)—react more slowly, with B showing slightly higher expansion than P [9,12,13]. The concrete mix design for the samples are summarized in Table 1. Prismatic specimens of dimensions 25 × 25 × 100 mm³ were produced using Portland cement CEM I 42.5 N (alkali content of 0.79 mass-% Na_2_O equivalent) and subjected to a standardized ASR protocol (SIA MB 2042). Following an initial curing period at 100% RH and 20°C for 24 hours, the specimens were demolded and immersed in an alkaline solution (0.3 M KOH, 0.1 M NaOH) at 40°C. Periodic measurements—including mass changes, length changes, tomography, and mechanical testing—were conducted over 1.5 years, with the samples then kept immersed for an additional year. Specimens with U aggregates consistently exhibited the highest ASR-induced expansion, while those with P aggregates showed the lowest, and B aggregates displayed intermediate behavior—reflecting their inherent mineralogical reactivity.

**Table 1:** Mix composition of the specimens in units of kg ⋅ m^-3^.

Specimen label	Cement I 42.5N	Aggregates sieve size			Deionized water	NaOH
		0-4 mm	4-8 mm	8-11.25 mm		
U, B, P	450	659	412	576	225	4.9

For the present study, one sample from each type was arbitrarily selected and trimmed to a smaller cross-section of 18 × 18 mm^2^ to facilitate detailed analysis. The samples were dried at 50°C for 72 hours. Once dried, we carefully wrapped each sample in multiple layers of aluminum foil to prevent rehydration and then the acquired reference neutron tomogram, to be considered as dry state or reference state. Next, we immersed the samples in deionized water for 72 hours to simulate a wetting cycle. After rehydration, the specimens were wrapped again to preserve their moisture content during a second round of neutron tomography, which allowed us to assess the structural changes induced by water absorption. Considering the specimens’ relatively small cross-section (18 × 18 mm²) and their extensive, pre-existing ASR-induced crack networks, the 72-hour immersion period was chosen to simulate a significant wetting event. This duration was deemed sufficient to allow for substantial water ingress directly into the primary cracks of both the cement paste and aggregates. While complete saturation of the entire fine pore matrix may not be achieved in this timeframe, the objective was to ensure sufficient water was available to the ASR products within the cracks to trigger an expansive response. The subsequent imaging confirmed this widespread water infiltration into the crack networks of both phases, as shown in Fig 3c.

**Fig 2:**
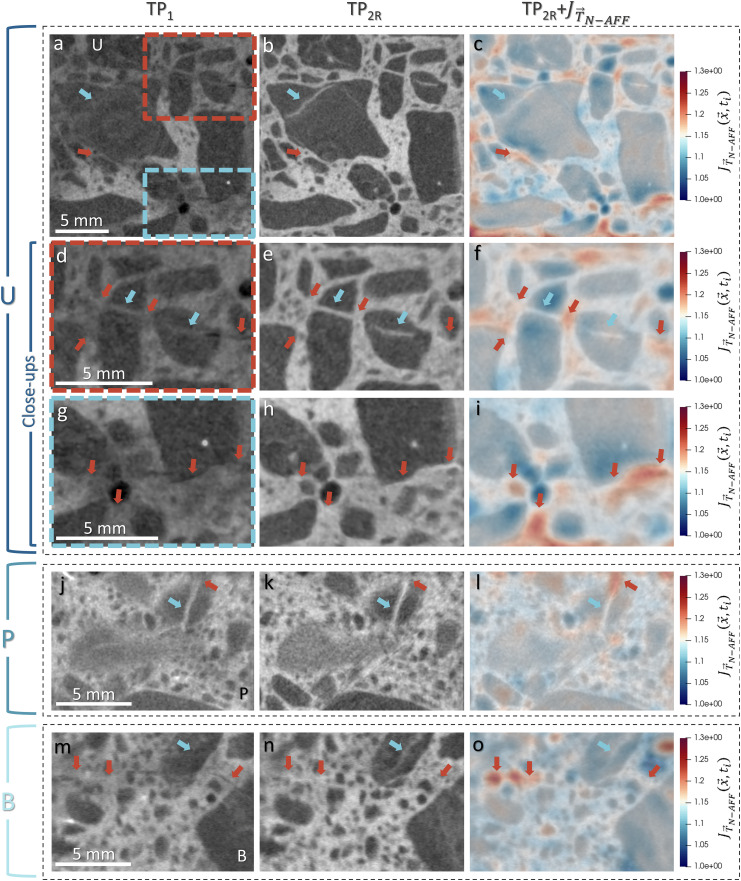
Neutron tomographic slices of three ASR‐damaged concrete samples,labeled U, P, and B, both before (dry state) and after (wet state) water exposure, alongside the corresponding local volumetric strain maps given by ${𝐉}_{{\vec{𝐓}}_{𝐍-AFF}}(\vec{𝐱},𝐭)$. Each sample is presented in a separate dotted‐outline block: **(a–i)** sample U, **(j–l)** sample P, and **(m–o)** sample **B.** Blue arrows highlight ASR‐induced cracks within aggregates, whereas orange arrows indicate cracks within the cement paste. The increase in brightness from dry to wet states confirms water uptake, evident both in aggregate cracks and in the surrounding cement paste. However, the strain maps reveal minimal expansion around cracks in the interior of aggregates, regions typically associated with crystalline ASR products, indicating that these products remain structurally stable despite moisture ingress. In contrast, cracks at aggregate boundaries and in the cement paste, where amorphous ASR products predominantly accumulate, exhibit significant local expansion, a finding which supports the hypothesis that amorphous phases are highly susceptible to swelling when exposed to water. “TP_1_” refers to the reference (dry) timepoint, while “TP_2R_” denotes the second timepoint (wet state) after non-affine registration.

### 2.2 Model samples

In addition to the concrete samples, we prepared a dedicated model system to simulate non-expanding cracks and validate our non-affine local registration method. This system allowed us to confirm that our technique can identify the absence of local expansion, even in the presence of neutron imaging noise and several other artifacts and the local intensity variation due to water addition to the system. We fabricated two model samples using aluminum and copper blocks with a 9.5 × 4.5 mm² cross-section with 200 microns thick with horizontal and vertical grooves cut using a diamond saw cutter. The blocks were then placed in a quartz cuvette with a 10 × 5 mm² cross-section and secured at the base with a drop of UV-curable glue to ensure stability during imaging. Similar to the concrete samples, this model system was imaged in two conditions: once in a dry state, and again after the cuvette was filled with water—filling the grooves and the surrounding air space. This idealized model system was specifically designed to validate a core assumption of our analysis: that the Mutual Information (MI) registration metric can distinguish image intensity variations—caused by the high neutron attenuation of water—from actual geometric changes in a known dimensionally stable material. We acknowledge that real cracks in cementitious systems are chemically and mechanically active and can exhibit behaviors like creep or diffuse swelling not captured by this simplified model system. The purpose of this validation was therefore not to mimic these complex phenomena, but to isolate and confirm the registration algorithm’s performance against the primary imaging challenge of large, non-linear intensity changes caused by water.

### 2.3 Neutron tomography

Neutron tomography was performed at the NEUTRA thermal neutron [19] imaging beamline at the measuring position 2 (L/D = 365). Radiographs were acquired using a CCD camera coupled to a 30 μm thick Gadox scintillator (RC TriTEC, Teufen, Switzerland) over a 70 × 70 mm² field of view resulting in a pixel size of 32.3 μm, with 625 projections (each with 80 s exposure time) uniformly distributed over 360°. The imaging sequence began with dark-current and open-beam radiographs for normalization, followed by black-body radiographs obtained using a 5-mm thick aluminum frame with a 3 × 8 grid of 2 mm cylindrical ^10^B_4_C inserts [20]. This setup allowed us to correct for the sample and background scattering. Finally, tomographic reconstructions—including normalization and scattering corrections, white spot and ring artifact removals—were performed using MuhRec software [21] based on a standard parallel beam filtered back projection algorithm.

### 2.4 Image registration for expansion analysis

Details on the image registration workflow are provided in the previous works [9,12,13]; here we only briefly summarize the key aspects. The goal of image registration is to precisely align the images so that any changes observed over time (between the two images) can be confidently attributed to true structural alterations, rather than misalignment or pure intensity changes due to water uptake. During registration we assume that the moving image (i.e., wet state), $I_{m}\left(\vec{x}\right)$, is related to the fixed image (i.e., dry state), $I_{f}\left(\vec{x}\right)$, by a displacement field $\vec{u}(\vec{x})$, with $\vec{x}$ being the spatial coordinate in $R^{\text{3}}$, such that:


$$\begin{array}{c} I_{m}\left(\vec{x}+\vec{u}(\vec{x})\right)=I_{f}\left(\vec{x}\right) \end{array}$$

In this framework, the displacement field $\vec{u}(\vec{x})$ becomes the core unknown we aim to determine for the quantitative analysis. We introduce a transformation function $T(\vec{x};\theta )$ that maps each point $\vec{x}$ in the moving image to a corresponding point in the fixed image. We can describe this as:


$$\begin{array}{c} \vec{T}\left(\vec{x};\theta \right)=\vec{x}+\vec{u}(\vec{x}) \end{array}$$

Where θ encapsulates the parameters governing $\vec{u}(\vec{x})$. The image registration process then seeks the θ that best aligns the two images according to our chosen similarity metric (e.g., mutual information, MI). By finding $\vec{u}(\vec{x})$, we effectively capture how each voxel in the moving image shifts to match the fixed image. We implemented our registration using the SimpleElastix library (an extension of Elastix integrated with SimpleITK) [22], which supports a range of transformation models from rigid and affine to non-affine (deformable) transformations.

#### 2.4.1 Mutual information as a robust similarity metric.

To quantify image similarity, we used Mutual Information (MI) as our cost function. MI is particularly well suited for our study because the reference tomogram and the post-water exposure tomogram can exhibit local, non-linear intensity changes due to water infiltration. Unlike correlation-based metrics that assume a linear relationship between voxel intensities, MI evaluates the statistical dependency between images by analyzing their joint intensity distributions. This means that even when the intensities differ significantly—as in the case of images behaving like different modalities—MI reliably captures true mechanical displacements rather than misinterpreting local intensity variations as structural changes. This robustness is critical for ensuring that our registration process accurately reflects real deformations in the concrete samples, regardless of the intensity shifts introduced by water. Mathematically MI is formulated as:


$$\begin{array}{c} MI\left(I_{f},I_{m};\vec{u}\right)=\sum_{s\in S_{f}}\sum_{t\in S_{m}}p\left(s,t;\vec{u}\right)log\frac{p\left(s,t;\vec{u}\right)}{p_{f}(s;\vec{u})p_{m}(t;\vec{u})} \end{array}$$

Where $S_{f}$ and $S_{m}$ are the sets of voxel intensity values in the fixed and moving images, respectively, and the probability density functions $p_{f}(s;\vec{u})\;$and $p_{m}(t;\vec{u})$, and the joint distribution $p\left(s,t;\vec{u}\right)$ are estimated using B-spline Parzen windowing.

#### 2.4.2 Transformation models.

As mentioned briefly above, the transformation function $T\left(\vec{x};\theta \right)$, maps points from the moving image to the same structures in the fixed image. For a rigid-body transformation, we have:


$$\begin{array}{c} {\vec{T}}_{RIG}\left(\vec{x};\theta \right)=R\left(\theta_{r}\right)\vec{x}+t \end{array}$$

Where $R\left(\theta_{r}\right)$ is the rotation matrix defined by the rotation angles and t is the translation vector. The registration process seeks the parameters θ that maximize $MI{(V}_{f},V_{m}({\vec{T}}_{RIG}\left(\vec{x};\theta \right)))$. The main goal of this registration is to correct for any misalignment introduced when the sample is removed from the imaging setup for water exposure and then repositioned for the subsequent tomography session.

Next, we used affine transformation to capture global deformations:


$$\begin{array}{c} {\vec{T}}_{AFF}\left(\vec{x};\theta \right)=A(\theta )\vec{x}+b \end{array}$$

Where A(θ) is a 3 × 3 matrix incorporating rotations, scalings, and shearings, and b is the translation vector. This model, with 12 degrees of freedom, allows us to estimate overall volumetric changes.

Both rigid and affine models assume constant local strain and cannot capture spatially varying deformations locally. Therefore, we employ a non-affine, deformable registration using B-spline functions:


$$\begin{array}{c} {\vec{T}}_{N-AFF}\left(\vec{x};\theta \right)=x+\sum_{i}\theta_{i}B_{i}(\vec{x}) \end{array}$$

Where $\theta_{i}$ are the displacements at the control points and $B_{i}(\vec{x})$ are the corresponding B-spline basis functions. Our registration framework formulated the alignment task as an optimization problem where we maximize the MI between the fixed and transformed moving images. We used an Adaptive Stochastic Gradient Descent (ASGD) optimizer [23], which efficiently navigates the large parameter space and adapts its learning rate to handle the non-convex, intricate cost function landscape.

#### 2.4.3 Computing local and global deformations.

A key output of the registration is the determinant of the Jacobian matrix, $J_{{\vec{T}}_{N-AFF}}(\vec{x},t)$, which quantifies local volume ratio associated with the transformation and is directly related to the volumetric strain ($\varepsilon_{v}=J_{{\vec{T}}_{N-AFF}}\left(\vec{x},t\right)-\text{1}$). Specifically, a determinant greater than one indicates local volume increase, less than one indicates local volume decrease, and a value equal to 1 means local volume preservation. For the affine transformation, this quantity represents the overall/global volume change of the analyzed volume, whereas for non-affine transformation it captures spatially varying local volume changes.

## 3 Results

Fig 2, shows the neutron tomogram slices before (i.e., dry state) and after (i.e., wet state) non-affine image registration and the superimposed scalar map of the $J_{{\vec{T}}_{N-AFF}}(\vec{x},t)$. The three concrete samples studied are labelled as U, P, and B, each presented in the three distinct blocks marked by a dotted rectangle in Fig 2, according to the aggregate types used in their production (as introduced in the Section 2.1). Some of the ASR induced cracks, in different samples are highlighted by blue and orange arrows, inside of the aggregates and in the cement paste, correspondingly. Comparing the tomogram slices before and after the water exposure, it is observed that the uptake of water is evident withing the aggregate cracks and also the cement paste manifested by increased brightness. Nevertheless, the local volume change maps, $J_{{\vec{T}}_{N-AFF}}(\vec{x},t)$, visually indicate that the most pronounced expansion appears to be concentrated around the cracks in the cement paste. Based on previous research and existing knowledge in the field of ASR [12,24], we assume that the ASR products in the cement paste cracks are typically amorphous, while those within the interiors of aggregates are mostly crystalline. Nevertheless, the ASR products in the aggregates can also be amorphous, depending on factors such as the age of the ASR, rock mineralogy, rock size, and the permeability of the aggregate to the pore solution, depending on the number of openings and cracks. Thus, slight expansions observed in the cracks within the aggregates might be due to the presence of some amorphous products within the aggregates. Furthermore, this could also be associated with the expansion capability of some crystalline phases [4], or simply because the opening of cracks in the cement paste is mechanically linked to those in the aggregates. Importantly, the general pattern of behavior is consistent across the three sample types, despite their known differences in ASR reactivity, suggesting a common underlying mechanism.

**Fig 3:**
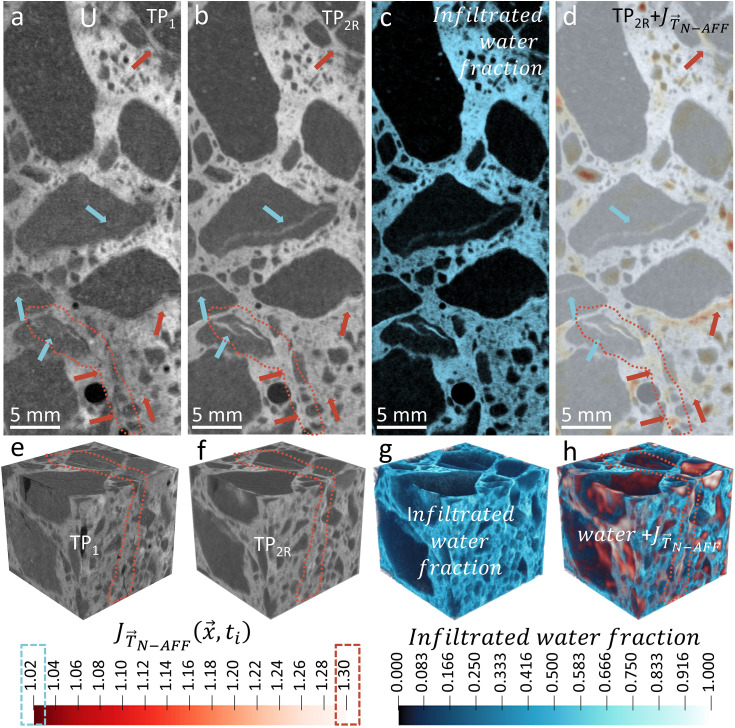
Neutron tomographic views of sample U, which contains the most reactive aggregates, illustrating its response to water exposure. **(a, b)** Tomogram slices acquired before (TP_1_) and after (TP_2R_) water infiltration, highlighting changes in brightness that indicate moisture ingress. **(c)** Relative water density map derived from neutron attenuation, revealing the distribution of infiltrated water within both the aggregates and the cement paste. **(d)** Two-dimensional local volumetric expansion map ${𝐉}_{{\vec{\text{T}}}_{\text{N}-\text{AFF}}}(\vec{\text{x}},\text{t})$, showing that the most pronounced deformations occur around cracks intersecting the cement paste and at aggregate–paste boundaries. **(e, f)** Three-dimensional renderings of the dry (TP_1_) and wet (TP_2R_) states, respectively. **(g)** The spatial distribution of infiltrated water fraction, illustrating how water permeates cracks in both the aggregates and the cement paste. **(h)** A three-dimensional representation of the local volume change shows that expansions are concentrated in paste-adjacent cracks, a finding consistent with the hypothesized susceptibility of amorphous ASR products to expanding in the presence of moisture. Overall, these observations reinforce the selective expansion mechanism. “TP_1_” refers to the reference (dry) timepoint, while “TP_2R_” denotes the second timepoint (wet state) after non-affine registration.

Fig 3 shows results similar to those in Fig 2, with a particular focus on sample U, which contains highly reactive aggregates. The ortho-slices of the tomogram clearly show relative changes in water content (Fig 3c), as indicated by variations in neutron attenuation before and after water exposure. Water infiltrates both the cement paste and the cracks within the aggregates. We can observe a substantial water penetration into the visible crack networks of aggregates (Fig 3c and g), making water accessible to ASR products there. However, the 2D local volume change map (Fig 3d) and its 3D representation (Fig 3h) visually indicate that the most significant expansion is concentrated around the cracks in the cement paste and at the interfaces between aggregates and paste.

**Fig 4:**
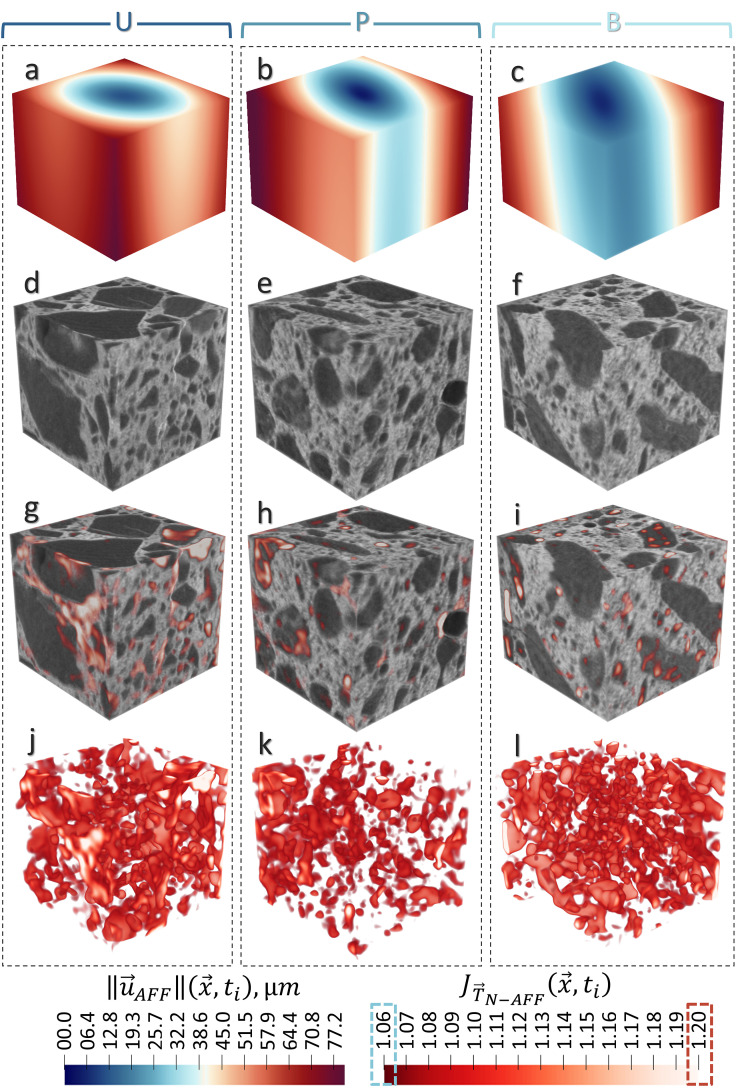
Global and local deformations observed in the three ASR‐damaged concrete samples (U, P, and B), each framed by a dotted outline in each column. **(a–c)** The 3D norm (magnitude) of the affine displacement field, $\left\|{\tilde{\vec{𝐮}\;}}_{\text{Aff}}(\vec{\text{x}},\text{t})\right\|$, illustrates overall expansion in each sample. Sample U (**a**) exhibits a relatively uniform lateral expansion, whereas samples P (**b**) and B (**c**) display less symmetrical deformation. The global volumetric strain, estimated from ${𝐉}_{{\vec{\text{T}}}_{\text{AFF}}}(\vec{\text{x}},\text{t})$, confirms that sample U undergoes the highest total strain (0.95%), followed by P (0.84%) and B (0.65%), in agreement with their known alkali-silica reactivity. **(d–l)** 3D tomograms of all samples before and after water exposure, superimposed with thresholded local volumetric strain fields ${𝐉}_{{\vec{\text{T}}}_{\text{N}-\text{AFF}}}(\vec{\text{x}},\text{t})$ between 1.06 and 1.2, highlight the most pronounced expansions.

Fig 4 presents both global and local deformations observed in all three samples. The global displacement is visualized by the 3D norm of the affine deformation field, $\left\|{\tilde{\vec{u}\;}}_{Aff}(\vec{x},t)\right\|$, shown in panels (a), (b), and (c). Because the determinant of the affine transformation function, $J_{{\vec{T}}_{N-AFF}}(\vec{x},t)$, yields a single volumetric strain value for global changes, rather than a spatially resolved map. For sample U (panel a), the deformation field reveals a uniform lateral expansion, while in sample P (panel b) and sample B (panel c), expansion is also present but less symmetrical. These deviations from uniform expansion likely stem from differences in aggregate distribution or crack network orientation. The quantified global volumetric strain, estimated from $J_{{\vec{T}}_{N-AFF}}(\vec{x},t)$, confirms that sample U undergoes the highest total volumetric expansion (i.e., strain) at 0.95%, followed by sample P (0.84%) and sample B (0.65%). This trend aligns with previous findings on alkali-silica reactivity, where sample U (sedimentary rock aggregates) exhibited the highest expansion after 500 days of ASR exposure, reaching 0.55%, whereas the granitic aggregates in samples P and B, known for their slower reactivity, expanded to only 0.35% and 0.29%, respectively [9,12,13]. We should consider that the volume of interest analyzed here may not be fully representative of the entire specimen, although the expansion amounts match those measured in representative volumes (whether by design or by coincidence). Nevertheless, the more important point to highlight here is that, regardless of any pattern of behavior, a clear global expansion is occurring in the samples as a result of water exposure.

When analyzing local deformations, tomograms of the samples before and after water exposure were overlaid with thresholded local volumetric strain fields $J_{{\vec{T}}_{N-AFF}}(\vec{x},t)$, (set between 1.06 and 1.2) to highlight the most pronounced expansions (panels d–l). The threshold range 1.06–1.2 was selected for two reasons. The lower bound was chosen conservatively based on the validation-based error analysis, since it lies well above the maximum background deviations observed in the control experiments (≤ 1.009 in the real control and ≤ 1.0012 in the phantom). The upper bound was introduced to focus the visualization on the dominant range of meaningful local expansions and to avoid a small number of extreme values from compressing the contrast of the main deformation field. A limited threshold-sensitivity evaluation further showed that the principal spatial localization of the expansion hotspots remained unchanged when nearby threshold values were used. Full details of the validation-based error assessment and threshold evaluation are provided in the Supplementary Information. While local expansion is observed throughout the samples, a visual assessment of the strain maps indicates that the most significant local expansions are concentrated in regions where amorphous ASR products are expected to be found—specifically, in the cracks within the cement paste and along the interfaces between the aggregates and the paste. The interconnected network of local volumetric strains reveals a complex expansion system within the concrete, where deformations in one region can influence others across the material. These deformation patterns are not simply a function of aggregate type or reactivity.

The reliability of the registration workflow was validated using dimensionally stable model systems and a digital phantom, for which water addition changes image intensity but does not produce true structural deformation. In these control cases, the non-affine registration did not generate systematic false-positive expansion in the water-filled regions, and only minor background deviations were observed, confirming that the method can distinguish intensity changes caused by moisture from actual geometric changes. These results support the interpretation that the deformation fields observed in the ASR-damaged concrete samples predominantly reflect real local structural changes rather than artifacts induced by neutron attenuation or registration bias. Full details of the validation procedure, control systems, phantom analysis, and error assessment are provided in the Supplementary Information.

## 4 Discussion

### 4.1 Context from previous meso-scale and micro-scale investigations

Previous petrographic and microscopy-based investigations had already shown in two-dimensional sections that ASR products can occur not only within reactive aggregates, but also at aggregate-paste interfaces and within cracks extending into the surrounding cement paste [25–27]. Building on this background, our earlier studies [9,12,13], based on X‐ray tomography analysis also confirmed that ASR products do not remain confined to the aggregates where they originate; instead, they migrate into the surrounding cement paste, driving significant structural and volumetric changes [2]. Our analysis revealed that local volumetric expansion, quantified via the determinant of the Jacobian from non‐affine image registration, $J_{{\vec{T}}_{N-AFF}}(\vec{x},t)$, frequently aligns with crack locations. This correlation underscores how crack formation and propagation contribute to global volumetric expansion [9,12,13]. Moreover, through image segmentation, we quantified the volume fractions of both ASR cracks and reaction products within aggregates and in the cement paste. Our results demonstrated that while cracks generally occupy a slightly larger fraction within aggregates, ASR products can accumulate equally or even slightly more in the cement paste over time. These findings highlight that although ASR initiates in aggregates, its progression into the cement matrix can be just as consequential.

The chemical and microstructural analyses have revealed notable differences between the ASR products [10]. Amorphous ASR products—often found at aggregate boundaries or extruding into the paste—consist mainly of Q^3^-sites (Si-tetrahedra with three bridging oxygen atoms typical for a layered structure) with a secondary amount of Q^2^-sites (Si-tetrahedra with two bridging oxygen atoms typical for a chain structure), as shown by Raman microscopy and 29Si MAS NMR [3]. In contrast, the crystalline ASR products, typically forming deeper within aggregates, are also dominated by Q^3^-sites but contain a smaller share of Q^2^-sites. The amorphous products, particularly those with a higher proportion of Q^2^-sites, feature larger interstitial spaces and a more open network than the denser, more ordered crystalline structures. Although the average Ca/Si ratio is very similar for both types, the (Na + K)/Si ratio varies more—most notably, the Na/K ratio is higher in amorphous ASR products. This higher proportion of Q^2^-sites, combined with the increased Na/K ratio, suggests that amorphous ASR products are more prone to water uptake and expansion. The larger interstitial spaces facilitate moisture absorption, and because sodium ions are smaller and more mobile than potassium ions, sodium-rich silicate phases tend to be more soluble and exhibit a stronger affinity for water [2]. This increased water uptake likely contributes to greater expansion in amorphous ASR products.

Supporting this differentiation, the in-situ 3D micro-XRD and micro-tomography investigations provided direct evidence that crystalline ASR products exhibit minimal expansion upon water uptake [4]. By extracting ASR-affected aggregate fragments and subjecting them to controlled humidity variations, we observed that the ASR products within aggregates were predominantly nano-crystalline, with distinct basal spacings and a heterogeneous spatial distribution. While some phases displayed slight increases in basal spacing under humid conditions—suggesting localized micro-expansion—these changes did not translate into significant macroscopic swelling. This finding aligns with previous studies indicating that crystalline ASR products, due to their rigid and stable structure, do not undergo substantial volumetric changes in response to moisture uptake. Their inability to absorb significant water reinforces the notion that amorphous ASR products are primarily responsible for the expansive pressures observed in ASR-induced damage [4].

At the very early stages, the location and type of ASR product formation are crucial in determining how damage initiates and evolves. A sequence of reactions leads to the accumulation of amorphous ASR products in confined spaces within aggregates, generating stress. When this stress exceeds the tensile strength of the aggregate, cracks initiate. Leemann’s microstructural analysis demonstrated that, upon cracking, amorphous ASR products immediately intrude into the new crack space, often forming a “plug” at the aggregate-cement paste interface, indicating that the reaction products were already under pressure [3]. As cracks propagate, the dynamics evolve: amorphous ASR products continue to migrate outward, while over time, crystalline ASR phases such as shlykovite and related silicate phases begin to form within the cracks. Emerging evidence points to precipitation (solidification) pressure as the key mechanism driving this process. A recent study by Leemann et al. [24] used a Surface Forces Apparatus to simulate the confined precipitation of ASR products. Their experiments demonstrated that as dissolved silica polymerized and solidified within a confined space, it exerted pressures between 6 and 13 MPa—sufficient to fracture most siliceous aggregates. This indicates that the act of ASR product formation itself generates stress that forces the aggregate apart. Once cracks form, continued accumulation of ASR products (sometimes referred to as mass accumulation) further contributes to expansion, although the contribution from later-forming crystalline phases, which grow through crystallization pressure, remains secondary to the initial expansion driven by amorphous ASR products.

### 4.2 Interpretation of moisture-induced strain fields in the current study

The current study provided direct visual insights about the moisture response of the already formed ASR products as a function of their spatial positioning. Using neutron tomography and image registration techniques, we generated spatially-resolved maps of moisture-induced expansions in concrete specimens that were heavily damaged by ASR. The observed expansions are not uniform but highly localized, with the most significant local deformations appearing in the cracks of the cement paste, regions where amorphous ASR products are most likely to be found, while water penetrates into the cracks within both the cement paste and the aggregates. In contrast, the regions dominated by crystalline ASR products, typically within larger aggregate cracks, show lesser expansion. This observation is critical for structural assessment; it suggests that water ingress into the paste crack networks can trigger local expansions, leading to renewed damage propagation. The global volumetric strains induced by a single 72-hour wetting cycle are remarkably high, with an average of 0.81 ± 0.15% across the three samples. While these laboratory values are not directly translatable to field performance, they serve as a clear indication of the material’s potential for reintroduced expansion. This insight is useful for practical engineering assessment.

It is important to acknowledge that the comparative analysis in this study is based primarily on the visual interpretation of the volume-change maps. While the present results confirm the promise of this image-based approach for revealing spatially localized moisture-induced deformation in ASR-damaged concrete, they should be regarded as preliminary. A more comprehensive follow-up study involving a larger number of samples and more extensive statistical analysis will be the necessary next step to evaluate the robustness and generality of the observed patterns. Such work, potentially including more strongly quantified region-based analysis, could also provide more direct input for numerical models.

## 5 Conclusions

Our study provides new insights into the moisture-driven expansion of ASR products, highlighting the role of their type (i.e., amorphous or more structured) in determining the local expansion behavior. Our analysis provides compelling visual evidence that while water penetrates cracks throughout the concrete mesostructure, the most significant moisture-induced local expansions are concentrated in the cement paste’s crack network. These regions are known to host amorphous ASR products, suggesting their higher expansive potential is a primary driver of damage upon re-wetting. ASR products within aggregates, often crystalline, appear to undergo less pronounced expansion. This behavior was consistent across all three sample types, indicating it is a consistent qualitative pattern across the three specimens analyzed. Our work successfully demonstrates the power of neutron tomography with strain mapping as a non-destructive method to track and visualize these complex chemo-mechanical interactions *in-situ*. An overall global expansion was also observed in all the specimens. The similar behavioral pattern observed in all the samples confirms a plausible shared mechanism across this sample set in response to the moisture/water. Although only one specimen per aggregate class, and only a small sub-volume of each was analyzed, all three specimens showed a similar, but broader validation is needed before generalizing these result. The key conclusions include:

- **Higher expansive potential of amorphous ASR products:** Our analysis, based on the interpretation of quantitative local volumetric strain maps, indicates that while water penetrates cracks in both cement paste and aggregates, the patterns of local expansion appear more significant in the cement paste’s cracks, regions where amorphous ASR products are likely to reside. ASR products within the aggregates, which are often predominantly crystalline, appear to undergo less pronounced local expansion upon water exposure under the conditions of this study.
- **Interconnected Expansion Network:** The local volumetric strain maps reveal a complex, interconnected network of deformations throughout the concrete that could lead to a global expansion.
- **Consistent Behavior Across Studied Aggregate Types:** The three specimen types, which feature aggregates with different ASR reactivities, show similar deformation patterns. This could indicate that the observed behavior is not merely a function of aggregate type or reactivity, but rather reflects a general characteristic of ASR-affected concrete.
- **Neutron Tomography as a Non-Destructive Monitoring Technic For ASR Reseacrh:** Although neutron imaging has been used to study moisture transport and durability processes in cementitious materials, its application to moisture-induced strain development in ASR-damaged concrete remains limited. The present study demonstrates that neutron tomography combined with advanced image registration can non-destructively visualize and quantify moisture distribution and the associated structural changes in ASR-damaged concrete without the use of contrast agents or other complex methods.

**Outlook:** This study marks an important step forward in understanding the complex interplay between ASR products and moisture. Future research should extend these methodologies to a broader range of concrete compositions and environmental conditions, further refining predictive models and guiding the development of more durable concrete formulations.

**Validation of the local volume change analysis:** S1 Fig Presents the results from the validation step designed to assess the reliability of our registration method in detecting true deformations in concrete samples. To achieve this, we used model systems that are known a priori to remain dimensionally stable upon moisture exposure. These model systems, consisting of copper and aluminum blocks with engraved horizontal and vertical grooves, were imaged using neutron tomography both in their dry state (reference) and after water was introduced. The grooves serve as artificial cracks that should remain unchanged, providing a controlled environment to test whether the method introduces false expansion artifacts. Validating our registration method using a rigid sample is a robust approach for establishing a baseline in our study. In concrete, crack opening behaves much like a rigid body simply separating—rather than deforming elastically—so simulating a crack with a groove in a rigid sample could be considered a valid comparison, although much simpler system than the crack network in a concrete structure.

S1 Fig a shows the model setup within a quartz cuvette in its dry state. After adding water, the neutron projection (S1 Fig b) shows the system was sealed with aluminum tape, ensuring consistent humidity conditions during imaging. The tomographic slices of the samples before and after water exposure are presented S1 Fig f-g and S1 Fig k-l along with the 3D rendering of the block (S1 Fig e-d) and the water filling the cuvette gaps and grooves (the blue-colored block of water). After non-affine registrations, the $J_{{\vec{T}}_{N-AFF}}(\vec{x},t)$ was computed to ensure that mere intensity variations in the images—resulting from water addition—do not falsely register as local expansion in the $J_{{\vec{T}}_{N-AFF}}(\vec{x},t)$ analysis. The 3D $J_{{\vec{T}}_{N-AFF}}(\vec{x},t)$ renderings, in S1 Fig h-j and S1 Fig m, show that in regions surrounding the grooves (i.e., water-filled) there is no significant local expansion, except for minor false positives, as large streaks in the sharp edges, due to reconstruction artifacts (the dark groves created in the tomograms due to high neutron scattering by water and sampling artifact).

To mitigate such unwanted false positives, two potential improvements could be implemented in the future tests. First, reducing the free space available for water inside cuvette would help minimize water content and thus neutron absorption, preventing severe beam attenuation effects. Second, using deuterated water (D_2_O) instead of regular water, due to its significantly lower neutron attenuation, could substantially improve reconstruction fidelity, ensuring more accurate Jacobian field calculations. Despite these minor limitations, the validation results confirm that the registration approach remains robust, reliably detecting true deformations while minimizing misinterpretations caused by image intensity changes.

S2 Fig Uses the results in S1 Fig to provide a more detailed 2D analysis which closely examines the accuracy of the non-affine registration process. This focused assessment allows for a finer inspection of potential registration errors by comparing both real experimental data and a digital phantom designed to mimic our control system. To ensure precise evaluation, we selected a region of interest (ROI) that excludes reconstruction artifacts, minimizing distortions and isolating true registration performance. The upper panels (a-d) depict the actual model sample, where panels a and b show tomographic slices before and after water addition, respectively. The corresponding panels c and d illustrate the effects of non-affine registration, superimposed with the determinant of the Jacobian field, $J_{{\vec{T}}_{N-AFF}}(\vec{x},t)$. Given that water merely fills the space around the sample without inducing physical deformation, any detected local expansion in these regions would indicate a registration artifact rather than a real structural change.

Several factors contribute to these registration errors. In the real model sample (panel d), inherent tomographic noise—stemming from variations in neutron flux, detector sensitivity, or other systematic inconsistencies—can introduce uncertainties. In low-information regions or areas with high noise, the optimization process in registration may converge incorrectly, producing small, localized errors. Additionally, B-spline-based transformations, while effective for capturing non-linear deformations, have inherent limitations in perfectly modeling complex distortions when faced with artifacts.

To test this on a more idealized test case, in the lower panels (e-h) we show the same procedure applied on a digital phantom, replicating a similar setup with water introduced around the sample. This phantom allows us to evaluate the technique on images free from natural noise or imaging artifacts. Panels e and f present the phantom slices before and after water addition, while panels g and h show the registration results with the Jacobian map overlaid. Notably, the $J_{{\vec{T}}_{N-AFF}}(\vec{x},t)$ maps in panels c and g (both the real sample and phantom) exhibit no significant expansion, as expected. However, when applying a tighter upper-bound threshold to emphasize potential registration errors, we observed minor deviations. These background errors ranged between 1–1.009 for the real model sample and a narrower range of 1–1.0012 for the phantom, as visualized in panels d and h. Importantly, these errors do not cluster around the water-introduced regions, suggesting that they are not due to systematic biases in the registration process. Rather, the small deviations observed in the phantom likely originate from inherent numerical approximations and discretization effects within the registration algorithm. Although minor inaccuracies exist, their magnitude is far lower than the deformations expected from ASR-induced damage.

Despite these minor inaccuracies, our findings affirm that the MI-based non-affine registration technique remains highly robust in distinguishing actual structural deformations from intensity variations introduced by experimental conditions, such as water addition. Unlike traditional intensity-based metrics such as cross-correlation, which assume a direct linear relationship between pixel intensities, MI quantifies statistical dependency between images, making it insensitive to uniform intensity shifts. This is crucial in our study, where water significantly alters image intensity due to neutron attenuation but does not physically deform the sample. While MI itself does not directly encode spatial information, the B-spline transformation framework integrates spatial consistency, ensuring that localized displacements remain physically meaningful. A multi-resolution registration strategy (beginning with coarse alignment and progressively refining at finer scales) further enhances robustness by stabilizing the optimization process. As illustrated in S2 Fig (third row, panel l), the MI metric optimizes the spatial alignment between images by adjusting voxel positions in TP2 (post-water exposure) to maximize the mutual information with TP1 (pre-exposure). This ensures that true local non-affine deformations—if present—are accurately captured. Importantly, in regions where water appears and has no corresponding feature in the dry image, the algorithm correctly identifies them as new, unmatched regions rather than attempting to register them erroneously.

It is important to note, that this validation model does not encompass the full complexity of concrete’s porous structure or potential for non-rigid, diffuse swelling phenomena. This simplified model was created only to confirm that the registration method can differentiate image‐intensity changes from water addition and genuine geometric alterations in a dimensionally stable material. Therefore, the interpretation of strains in the concrete samples assumes that the observed deformations primarily relate to the opening/closing of cracks and local material expansion, phenomena the algorithm is designed to capture.

## Supporting information

S1 FigValidation of the registration method using model systems known to remain dimensionally stable under moisture exposure.(a) Photograph of the quartz cuvette setup in its dry state, containing copper and aluminum blocks with engraved horizontal and vertical grooves that act as artificial cracks. (b) Neutron projection after water addition, sealed with aluminum tape to maintain consistent humidity conditions during imaging. (c, d, e) Schematics of the model systems, illustrating the grooves and the arrangement of blocks within the cuvette. (f, g) Tomography slices of the copper block before and after water introduction, and (h, i, j) three-dimensional renderings of the corresponding ${\text{J}}_{{\vec{\text{T}}}_{\text{N}-\text{AFF}}}(\vec{\text{x}},\text{t})$ fields, indicating minimal false expansion around the water-filled grooves. (k, l) Tomography slices of the aluminum block before and after water addition, and (m) the 3D ${\text{J}}_{{\vec{\text{T}}}_{\text{N}-\text{AFF}}}(\vec{\text{x}},\text{t})$ map. In both copper and aluminum samples, the grooves remain dimensionally unchanged, verifying that intensity variations due to water infiltration do not spuriously register as expansion.(PNG)

S2 FigA focused 2D evaluation of the non-affine registration process, comparing a real model sample (upper panels, a–d) with a digitally simulated phantom (lower panels, e–h).To isolate genuine registration accuracy, a region of interest (ROI) excluding reconstruction artifacts was selected, ensuring minimal distortions. (a, b) Tomographic slices of the real model sample in its dry state (TP_1_) and after water addition (TP_2_), where water merely fills the space around the sample without inducing any true deformation. (c, d) Corresponding determinant-of-the-Jacobian ${\text{J}}_{{\vec{\text{T}}}_{\text{N}-\text{AFF}}}(\vec{\text{x}},\text{t})$ maps superimposed on the tomograms, illustrating that no significant local expansion occurs near the water-filled regions; minor deviations primarily stem from image noise and artifacts rather than systematic registration errors. (e, f) Equivalent slices for a digitally simulated phantom before and after water addition, serving as an idealized, noise-free baseline. (g, h) Registration results for the phantom, which confirm that only negligible background errors—falling in the range 1–1.0012—are detected, reinforcing the hypothesis that largest observed discrepancies in the real sample are artifact-driven. (i-l) These panels schematically illustrate how the mutual-information (MI)–based registration distinguishes real structural changes from intensity shifts between the dry (TP_1_) and wet (TP2) states. Panels (i–k) show how air is replaced by water in the upper region, altering image intensities due to neutron attenuation. In panel (l), we summarize how the registration algorithm applies the “image intensity conservation” assumption in the resulting registered image. A voxel at a given position of moving image (TP_2_) is assumed to be registered to the reference image while retaining the same intensity value at both time points (in the same modality images) in the ideal case. However, even in the case of non-linear local intensity variations, MI does not require the same numeric intensity values in both images, as it seeks a transformation that maximizes the statistical dependence (i.e., the joint probability distribution) of voxel intensities between the two images. Thus, the “conservation” means that the underlying structures in Image 1 correspond consistently to those in Image 2.(PNG)
